# Presence of Anti-Thyroid Antibodies Correlate to Worse Outcome of Anti-NMDAR Encephalitis

**DOI:** 10.3389/fimmu.2021.725950

**Published:** 2021-09-08

**Authors:** Zhongyun Chen, Yan Zhang, Lili Cui, Huijin Huang, Weibi Chen, Yingying Su

**Affiliations:** ^1^Department of Neurology, Xuanwu Hospital, Capital Medical University, Beijing, China; ^2^Institute of Sleep and Consciousness Disorders, Beijing Institute of Brain Disorders, Collaborative Innovation Center for Brain Disorders, Capital Medical University, Beijing, China

**Keywords:** anti-N-methyl-D-aspartate receptor encephalitis, anti-thyroglobulin antibody, anti-thyroperoxidase antibody, critically ill, outcome

## Abstract

**Objective:**

To investigate the characteristics and prognosis of anti-NMDAR encephalitis with the prevalence of anti-thyroid antibodies (ATAbs).

**Methods:**

The clinical data of anti-NMDAR encephalitis patients admitted to Xuanwu Hospital from January 2012 to August 2018 was prospectively analyzed, and the patients were followed up for 24 months.

**Results:**

A total of 120 patients were enrolled, of which 34.2% (41/120) were positive for ATAbs. The antibodies were more frequent in patients with severe disease compared to the non-severe group (51.4% *vs*. 25.6%, P=0.008). In addition, prevalence of ATAbs correlated with a higher incidence of disturbed consciousness, autonomic dysfunction, central hypoventilation and mechanical ventilation. The ATAbs-positive patients were also more likely to receive intravenous gamma immunoglobulin and immunosuppressor compared to the ATAbs-negative cases (P=0.006; P=0.035). Although the presence of ATAbs was associated with longer hospital stays and worse prognosis at 6 months (P=0.006; P=0.038), it had no impact on long-term patient prognosis. Positive status of anti-thyroglobulin antibody was an independent risk factor for worse prognosis at 6 months [odds ratio (OR)= 3.907, 95% CI: 1.178-12.958, P=0.026].

**Conclusion:**

ATAbs are prevalent in patients with anti-NMDAR encephalitis, especially in severe cases, and correlate with poor prognosis and impaired short-term neurological recovery.

## Introduction

Anti-thyroid antibodies (ATAbs), including anti-thyroglobulin antibody (anti-TgAb) and anti-thyroperoxidase antibody (anti-TPOAb), are pathological markers of autoimmune thyroid disease. ATAbs are frequently detected in central nervous system (CNS) autoimmune diseases such as neuromyelitis optica spectrum disorders (NMO-SD) (1, 2), multiple sclerosis (3) and autoimmune encephalitis (AE) (4, 5). However, the pathogenic role of ATAbs in CNS autoimmune disease remains unclear. It may be associated with increased susceptibility for CNS autoimmunity. Previous studies have reported that ATAbs are significantly elevated in AE; besides, patients with ATAbs are inclined to develop anti-neuronal immune responses and AE, indicating that CNS autoimmune disease and autoimmune thyroid disease may represent a pathogenic spectrum (4, 6). ATAbs have also been considered as initial diagnostic markers when clinically suspecting AE or autoimmune epilepsy (5, 7). In addition, ATAbs are also found to be associated with disease severity in NMO-SD (1, 2).

Anti-N-methyl-D-aspartate receptor (NMDAR) encephalitis is an AE characterized by the production of autoantibodies against antigenic nerve surfaces or synapses (8). Very few studies have reported the coexistence of ATAbs in anti-NMDAR encephalitis (6, 9–13), and their clinical and prognostic relevance in anti-NMDAR encephalitis has not been systematically evaluated. The aim of this study was to determine the correlation between ATAbs and various clinicopathological factors in a cohort of anti-NMDAR encephalitis patients.

## Methods

### Study Design and Participants

We prospectively recruited anti-NMDAR encephalitis patients from the Department of Neurology of Xuanwu Hospital, Capital Medical University between January 2012 and August 2018 based on the following inclusion criteria (1): age ≥14 years old (2), acute or subacute onset symptoms of encephalitis (< 3 months) (3), presence of abnormal behavior or cognitive dysfunction, speech dysfunction, seizures, movement disorders, decreased level of consciousness, autonomic dysfunction or central hypoventilation, or a combination of the above symptoms (4), positive anti-GluN1 NMDAR antibodies in the cerebrospinal fluid (CSF) and/or serum positivity, and (5) absence of viral encephalitis, brain tumors, metabolic diseases, drug poisoning, et al. The exclusion criteria were as follows (1): non-compliance with the treatment (2), presence of other autoimmune antibodies or neurological paraneoplastic antibodies (3), not the first onset of anti-NMDAR encephalitis (4), history of thyroid diseases (5), prior prescription, such as thyroid hormones, antithyroid drugs, β-blockers, etc. (6), without thyroid function test (7), lost to the 6-month follow-up. The patients were divided into the severe and non-severe disease groups based on whether they were admitted to the neurological intensive care unit (NCU). Patients with severe disease who were admitted to the NCU met at least one of the following criteria: respiratory failure requiring mechanical ventilation, impaired consciousness (GCS ≤ 12), or status epilepticus.

### Data Collection

Data regarding age of onset, sex, prodromal symptoms, clinical characteristics, CSF findings, brain magnetic resonance imaging (MRI) and electroencephalography (EEG) findings, treatment details and follow-up data were retrieved. The anti-NMDAR Encephalitis One-Year Functional Status (NEOS) score, including NCU admission, delayed treatment for more than 4 weeks, no improvement in clinical outcomes within 4 weeks, abnormal MRI, and white blood cell count of more than 20 cells/μL in CSF, was considered as a prognostic tool (14). Anti-GluN1 NMDA antibodies in the serum and CSF were measured using indirect immunofluorescence test (IIFT) kits (EUROIMMUN AG, Lübeck, Germany). Thyroid function was measured at 6 AM the day after admission using standardized radioimmunoassay kits. A patient was considered ATAbs-positive if the levels were >4 IU/ml for anti-TgAb or > 9 IU/ml for anti-TPOAb.

### Outcomes

All the patients were followed up by outpatient clinic visit or phone contact by resident doctors who are not aware of the study design at 6, 12 and 24 months after discharge. The modified Rankin Scale (mRS) was used to evaluate treatment effects and prognosis. Relapse was defined as the deterioration of previous symptoms or the appearance of new symptoms two months after stabilization (15). Favorable and unfavorable outcomes were defined as mRS scores of 0-2 and 3-6 respectively.

### Statistical analysis

Statistical analyses were performed using SPSS 20.0 (IBM Corporation, Armonk, NY, USA). Data are presented as mean ± SD, medians with the interquartile range (IQR) or counts (percentages), and compared by Student’s t test, Mann–Whitney U test, Pearson chi-square test or Fisher exact test as appropriate. Variables with P < 0.1 in the univariate analysis were included in multivariate logistic regression. A back-ward selection procedure was used for the screening of independent risk factors predicting the unfavorable outcomes at 6 months. P values < 0.05 were considered statistically significant.

## Results

### Patient Characteristics

**Figure 1:**
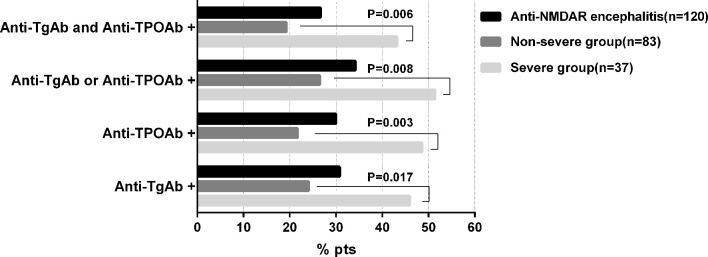
Prevalence of ATA positivity in Anti-NMDAR encephalitis patients. ATAbs, Anti-thyroid antibodies; anti-TgAb, anti-thyroglobulin antibody; anti-TPOAb, anti-thyroperoxidase antibody.

One hundred and twenty patients (67 males and 53 females, mean age 29.3 ± 13.4 years) diagnosed with anti-NMDAR encephalitis were enrolled (**Supplemental Figure 1**). The overall prevalence of ATAbs was 34.2%. As shown in **Figure 1**, the patients with severe disease had a significantly higher proportion of those positive for one or both ATAbs compared to that in the non-severe group.

### Demographic, Clinical Characteristics, and Prognosis of Patients With or Without ATAbs

Furthermore, patients with ATAbs had higher incidence of disturbed consciousness (63.4% *vs*. 36.7%, P=0.005), autonomic dysfunction (48.8% *vs*. 17.7%, P<0.001), central hypoventilation (34.1% *vs*. 15.2%, P=0.010), mechanical ventilation (34.1% *vs*. 15.2%, P=0.017) and NCU admission (46.3% *vs*. 22.8%, P=0.008) (**Table 1**). As shown in **Table 2**, patients with ATAbs had lower FT3 levels (P=0.012) and while the EEG features (epileptic discharges, slow activity, and others), MRI lesions, CSF findings (pressure of lumbar puncture, white blood cells and protein levels), and the serum and CSF antibody titers were similar between the ATAbs-positive and ATAbs-negative patients. The ATAbs-positive patients were also more likely to receive intravenous immunoglobulins (IVIG) and immunosuppressor treatment (P=0.006, P=0.035), and have longer hospital stays and worse neurological outcomes at 6 months (P=0.006, P=0.038) (**Table 1** and **Supplementary Figure 2**). There was no significant difference in the relapse and functional outcomes at 12 and 24 months in both groups (**Table 1**).

**Table 1:** Demographic, clinical characteristics and prognosis of anti-NMDAR encephalitis patients with or without ATAbs.

	Total (n = 120)	ATAbs negative (n = 79)	ATAbs positive (n = 41)	P value
Age at onset, y, mean ± SD	29.3 ± 13.4	28.8 ± 12.6	30.3 ± 14.9	0.516
Female, n (%)	53 (44.2)	32 (40.5)	21 (51.2)	0.262
Ovarian teratoma, n (%)	8 (6.7)	3 (3.8)	5 (12.2)	0.080
Prodromal symptoms, n (%)	63 (52.5)	41 (51.9)	22 (53.7)	0.855
Clinical manifestations, n (%)				
Mental behavior disorder	87 (72.5)	55 (69.6)	32 (78.0)	0.327
Epileptic seizure	78 (65.0)	51 (64.6)	27 (65.9)	0.888
Involuntary movement	56 (46.7)	34 (43.0)	22 (53.7)	0.269
Disturbance of consciousness	55 (45.8)	29 (36.7)	26 (63.4)	0.005
Cognitive impairment	37 (30.8)	20 (25.3)	17 (41.5)	0.069
Autonomic dysfunction	34 (28.3)	14 (17.7)	20 (48.8)	<0.001
Language impairment	27 (22.5)	14 (17.7)	13 (31.7)	0.082
Central hypoventilation	25 (20.8)	11 (13.9)	14 (34.1)	0.010
Mechanical ventilation, n (%)	26 (21.7)	12 (15.2)	14 (34.1)	0.017
NCU admission, n (%)	37 (30.8)	18 (22.8)	19 (46.3)	0.008
NEOS score, median (IQR)	2 (2,4)	2 (2,3)	3 (2,4)	0.190
Immunotherapy, n (%)				
Steroids	102 (85.0)	66 (83.5)	36 (87.8)	0.535
IVIG	70 (58.3)	39 (49.4)	31 (75.6)	0.006
Plasma exchange	22 (18.3)	11 (13.9)	11 (26.8)	0.083
Immunosuppressor	25 (20.8)	12 (15.2)	13 (31.7)	0.035
Outcome				
Length of NCU length of stay, n=37, days, median (IQR)	39.0 (23.0-73.0)	37.0 (17.0,70.0)	45.0 (24.0,82.0)	0.362
Hospital length of stay, days, median (IQR)	18.0 (13.0-37.5)	12.0 (12.0,27.0)	21.0 (15.0,58.5)	0.006
Relapse, n (%)	14 (11.7)	11 (13.9)	3 (7.3)	0.285
mRS>2 after 6 months, n (%)	18 (15.0)	8 (10.1)	10 (24.4)	0.038
mRS>2 after 12 months, n=101, n (%)	20 (19.8)	13 (19.7)	7 (20.0)	0.971
mRS>2 after 24 months, n=96, n (%)	9 (9.4)	5 (8.2)	4 (11.4)	0.601

ATAbs, Anti-thyroid antibodies; SD, standard deviation; IVIG, intravenous immunoglobins; mRS, modified Ranking scale; NCU, neurological intensive care unit; NEOS, the anti-NMDAR Encephalitis One-Year Functional Status.

**Table 2:** Auxiliary features of anti-NMDAR encephalitis patients with or without ATAbs.

	Total (n = 120)	ATAbs negative (n = 79)	ATAbs positive (n = 41)	P value
Thyroid function				
TSH, uIU/ml, median (IQR)	1.60 (0.94,2.57)	1.59 (0.91,2.48)	1.84 (1.16,2.91)	0.180
TT3, ng/ml, median (IQR)	0.83 (0.64,1.01)	0.87 (0.68,1.02)	0.79 (0.62,0.93)	0.051
TT4, ug/dl, median (IQR)	7.5 (6.1,9.0)	7.8 (6.1,9.1)	7.2 (6.1,8.3)	0.238
FT3, pg/ml, median (IQR)	2.7 (2.2,3.1)	2.8 (2.4,3.3)	2.58 (2.0,2.9)	0.012
FT4, ng/dl, median (IQR)	1.14 (0.98,1.27)	1.16 (0.98,1.28)	1.12 (0.93,1.23)	0.464
Anti-TgAb, IU/ml, median (IQR)	0.4 (0.02,14.1)	0.1 (0-0.4)	46.2 (12.4,77.0)	<0.001
Anti-TPOAb, IU/ml, median (IQR)	1.5 (0.5,24.3)	0.7 (0.3,1.5)	48.5 (22.7,82.6)	<0.001
EEG, n=94, n (%)				
Normal	9 (9.6)	7 (11.3)	2 (6.3)	0.431
Slow activity	50 (53.2)	35 (56.5)	15 (46.9)	0.378
Epileptic discharges	22 (23.4)	12 (19.4)	10 (31.3)	0.197
others	13 (13.8)	8 (12.9)	5 (15.6)	0.717
Cranial MRI, n, (%)				
Normal	45 (37.5)	30 (38.0)	15 (36.6)	0.881
Limbic lobe	43 (35.8)	26 (32.9)	17 (41.5)	0.354
Temporal lobe	31 (25.8)	18 (22.8)	13 (31.7)	0.290
Frontal lobe	27 (22.5)	17 (21.5)	10 (24.4)	0.721
Insular lobe	15 (12.5)	7 (8.9)	8 (19.5)	0.094
Parietal lobe	14 (11.7)	9 (11.4)	5 (12.2)	0.897
Occipital lobe	10 (8.3)	5 (6.3)	5 (12.2)	0.270
Brainstem	9 (7.5)	5 (6.3)	4 (9.8)	0.499
Basal ganglia	7 (5.8)	3 (3.8)	4 (9.8)	0.187
Diencephalon	4 (3.3)	2 (2.5)	2 (4.9)	0.605
CSF analysis				
Opening pressure, mmH_2_O, median (IQR)	180 (140,229)	180 (135,220)	190 (140,240)	0.740
WBC, ×10^6^/L, median (IQR)	19.0 (6.0,35.0)	19.0 (5.0,35.0)	19.5 (7.3,38.5)	0.611
Protein, mg/dl, median (IQR)	32.0 (21.0,44.5)	31.0 (21.0,45.0)	34.0 (19.3,44.8)	0.411
CSF NMDAR antibody titers, n (%)				
+	14 (11.7)	8 (10.1)	6 (14.2)	0.466
++	65 (54.2)	42 (53.2)	23 (56.1)	0.760
+++	41 (34.2)	29 (36.7)	12 (29.3)	0.415
Serum NMDAR antibody titers, n (%)				
-	64 (53.3)	43 (54.4)	21 (51.2)	0.738
+	18 (15.0)	11 (13.9)	7 (17.1)	0.647
++	32 (26.7)	22 (27.8)	10 (24.4)	0.685
+++	6 (5.0)	3 (3.8)	3 (7.3)	0.410

ATAbs, Anti-thyroid antibodies; anti-TgAb, anti-thyroglobulin antibody; anti-TPOAb, anti-thyroperoxidase antibody; CSF, cerebral spinal fluid; EEG, electroencephalography; IQR, interquartile range; FT3, free triiodothyronine; FT4, free thyroxine; MRI, magnetic resonance imaging; TT3, triiodothyronine; TT4, total thyroxine; TSH, thyroid stimulating hormone.

### Functional Outcome at 6 Months

Univariate analysis indicated that the older (mean: 37.9 *vs*. 27.8 years, P=0.003), mechanical ventilation (44.4% *vs*. 17.6%, P=0.011), NCU admission (61.1% *vs*. 25.5%, P=0.003), higher levels of NEOS scores (median: 4 *vs*. 2, P<0.001), and Anti-TgAb (55.6% *vs*. 25.6%, P=0.014) were associated with unfavorable outcomes at 6 months. Patients with unfavorable outcomes at 6 months also presented fewer prodromal symptoms (P=0.069), more disturbed consciousness (P=0.054), higher titers of Anti-TgAb (P=0.062) and more frequency receiving plasma exchange (P=0.074). Multivariate logistic regression analysis demonstrated that age at onset >31 years [odds ratio (OR)= 3.545, 95% CI: 1.070-11.738, P=0.048], NEOS score 4-5 (OR=9.660; 95% CI: 2.906-32.105, P<0.001), and anti-TgAb (OR=3.907, 95% CI: 1.178-12.958, P=0.026) were independent factors for the prediction of unfavorable outcomes at 6 months (**Table 3**).

**Table 3:** Univariate and multivariate logistic regression analyses of outcome at 6 months.

	Univariate analyses			Multivariate analyses	
	Favorable outcome at 6 months (n = 102)	Unfavorable outcome at 6 months (n = 18)	P value	OR (95%CI)	P value
Age at onset, y, mean ± SD	27.8 ± 11.7	37.9 ± 18.8	0.003		
Age at onset >31 years, n (%)*	35 (34.2)	11 (61.1)	0.031	3.545 (1.070-11.738)	0.038
Female, n (%)	43 (42.2)	10 (55.6)	0.291		
Ovarian teratoma, n (%)	6 (5.9)	2 (11.1)	0.412		
Prodromal symptoms, n (%)	50 (49.0)	13 (20.6)	0.069	–	–
Clinical manifestations, n (%)					
Mental behavior disorder	75 (73.5)	12 (66.7)	0.548		
Epileptic seizure	68 (66.7)	10 (55.6)	0.362		
Involuntary movement	46 (45.1)	10 (55.6)	0.412		
Disturbance of consciousness	43 (42.2)	12 (66.7)	0.054	–	–
Cognitive impairment	29 (28.4)	8 (44.4)	0.175		
Autonomic dysfunction	26 (25.5)	8 (44.4)	0.100		
Language impairment	21 (20.6)	6 (33.3)	0.233		
Central hypoventilation	19 (18.6)	6 (33.3)	0.157		
Mechanical ventilation, n (%)	18 (17.6)	8 (44.4)	0.011	–	–
NCU admission, n (%)	26 (25.5)	11 (61.1)	0.003		
NEOS score, median (IQR)	2 (2,3)	4 (3.5,4.3)	<0.001		
NEOS score 4-5, n (%)	21 (20.6)	13 (72.2)	<0.001	9.660 (2.906-32.105)	<0.001
Electroencephalogram, n=94, n (%)					
Normal	8 (7.8)	1 (5.6)	0.734		
Slow activity	18 (17.6)	4 (22.2)	0.644		
Epileptic discharges	42 (41.2)	8 (44.4)	0.795		
others	10 (9.8)	3 (16.7)	0.388		
Cranial MRI, n, (%)					
Normal	40 (39.2)	6 (33.3)	0.636		
Lesions in cerebral cortex	43 (42.2)	7 (38.9)	0.795		
Lesions in white matter	17 (16.7)	5 (27.8)	0.261		
CSF analysis					
Opening pressure, mmH_2_O, median (IQR)	180 (140,225)	210 (115,273)	0.628		
WBC, ×10^6^/L, median (IQR)	15.0 (6.5,33.5)	29.0 (3.5,47.3)	0.228		
Protein, mg/dl, median (IQR)	31.0 (21.0,43.0)	38.0 (19.8,53.0)	0.389		
CSF NMDAR antibody titers, n (%)					
+	12 (11.8)	2 (11.1)	0.937		
++	58 (56.9)	7 (38.9)	0.158		
+++	32 (31.4)	9 (50.0)	0.124		
Serum NMDAR antibody titers, n (%)					
-	52 (51.0)	12 (66.7)	0.219		
+	16 (15.7)	2 (11.1)	0.616		
++	28 (27.5)	4 (22.2)	0.644		
+++	6 (5.9)	0	0.291		
ATAbs					
Anti-TgAb +	27 (25.6)	10 (55.6)	0.014	3.907 (1.178-12.958)	0.026
Anti-TgAb titers, IU/ml, median (IQR)	0.2 (0-10.5)	6.2 (0.2-56.7)	0.062		
Anti-TPOAb +	29 (28.4)	7 (38.9)	0.372		
Anti-TPOAb titers, IU/ml, median (IQR)	1.4 (0.4-21.2)	1.5 (0.6-40.6)	0.513		
Anti-TgAb or Anti-TPOAb +	31 (30.4)	10 (55.6)	0.038		
Anti-TgAb and Anti-TPOAb +	25 (24.5)	7 (38.9)	0.203		
Immunotherapy, n (%)					
Steroids	88 (86.3)	14 (77.8)	0.352		
IVIG	58 (56.9)	12 (66.7)	0.437		
Plasma exchange	16 (15.7)	6 (33.3)	0.074	–	–
Immunosuppressor	19 (18.6)	6 (33.3)	0.157		
Outcome					
Length of NCU length of stay, n=37, days, median (IQR)	37.5 (21.0,73.0)	44.0 (24.0,82.0)	0.666		
Hospital length of stay, days, median (IQR)	18.0 (13.0,29.0)	32.5 (16.5,52.3)	0.121		
Relapse, n (%)	13 (12.7)	1 (5.6)	0.381		

*The optimal cutoff value of age to predict unfavorable outcome was 31 with a sensitivity of 61.1% and a specificity of 69.6%. ATAbs, Anti-thyroid antibodies; CSF, cerebral spinal fluid; CI, confidence interval; EEG, electroencephalography; IQR, interquartile range; IVIG, intravenous immunoglobins; MRI, magnetic resonance imaging; NCU, neurological intensive care unit; NEOS, the anti-NMDAR Encephalitis One-Year Functional Status; OR, odds ratio.

## Discussion

ATAbs are present in more than 10% of the healthy population (16), and the titer depends on age and sex. In the present study, the overall ATAbs positivity rate in patients with anti-NMDAR encephalitis was 37.1%, which is similar to that seen in other autoimmune diseases (17), but increased to 51.4% in the severe cases. Patients with ATAbs displayed higher rates of impaired consciousness, autonomic dysfunction, central hypoventilation and mechanical ventilation, all of which are typical features of severe anti-NMDAR encephalitis. Similar observations have been reported by Lin et al (9). In addition, the severity of NMO-SD is also associated with the presence of ATAbs (1). Elevated levels of ATAbs may lead to immune dysfunction in the brain by interacting with antibodies directed against neuronal surface antigens, which in can trigger a more aggressive autoimmune response against neurons. Therefore, it is reasonable to surmise that anti-NMDAR encephalitis patients with ATAbs may have a worse prognosis. The ATAbs-positive patients receiving aggressive immunotherapy show improved long-term prognosis. Interestingly, we identified anti-TgAb as an independent risk factor of poor short-term prognosis of anti-NMDAR encephalitis, although the positive rate of anti-TgAb in critically ill patients is lower than that of anti-TPOAb. The exact mechanisms remain to be unraveled.

Hashimoto’s encephalopathy (HE) has similar neurological and psychiatric symptoms as AEs, but can be distinguished from the latter on the basis of ATAbs (8). As the low specificity of ATAbs defining HE and their coexistence with other antibodies defining various AEs. It is possible that ATAbs may only be part of an episodic phenomenon triggered by anti-neuronal antibodies, which should be the treatment focus for patients exhibiting symptoms of immune-mediated encephalitis regardless of the presence of ATAbs. Since most ancillary tests for HE are non-specific, we also did not find specific features of anti-NMDAR encephalitis in the ATAbs-positive patients in the ancillary tests. Nevertheless, one study has reported that ATAbs-positive patients have more common limbic system lesions (9).

In conclusion, the prevalence of ATAbs is common in patients with anti-NMDAR encephalitis, especially in the severe cases. Presence of ATAbs is associated with worse short-term neurological recovery and may be need more aggressive immunotherapy.

## Data Availability Statement

The original contributions presented in the study are included in the article/**Supplementary Material**. Further inquiries can be directed to the corresponding author.

## Ethics Statement

The studies involving human participants were reviewed and approved by Ethics Committee of the Xuanwu Hospital. Written informed consent to participate in this study was provided by the participants’ legal guardian/next of kin.

## Author Contributions

ZC carried out the patients enrollment, statistical analysis and drafted the manuscript. HH carried out the patients enrollment and verification of data. WC and LC carried out the verification of data and statistical analysis. YZ and YS conceived of the study, and participated in its design and coordination and helped to draft the manuscript. All authors contributed to the article and approved the submitted version.

## Funding

This project was supported by the National Key Research and Development Program of China Research (2020YFC2005403) and by Beijing Municipal Administration of Hospitals Incubating Program (PX2020035).

## Conflict of Interest

The authors declare that the research was conducted in the absence of any commercial or financial relationships that could be construed as a potential conflict of interest.

## Publisher’s Note

All claims expressed in this article are solely those of the authors and do not necessarily represent those of their affiliated organizations, or those of the publisher, the editors and the reviewers. Any product that may be evaluated in this article, or claim that may be made by its manufacturer, is not guaranteed or endorsed by the publisher.

## Supplementary Material

Supplementary Figure 1 Patient enrollment flow chart.

Supplementary Figure 2 Relationship between ATAbs and prognosis at 6 months. ATAbs, Anti-thyroid antibodies; anti-TgAb, anti-thyroglobulin antibody; anti-TPOAb, anti-thyroperoxidase antibody.
