# Supplementary figures and images for: Presence of Anti-Thyroid Antibodies Correlate to Worse Outcome of Anti-NMDAR Encephalitis
Source: Front Immunol. 2021 Sep 8;12:725950. doi: 10.3389/fimmu.2021.725950 (PMC8456002; doi:10.3389/fimmu.2021.725950)

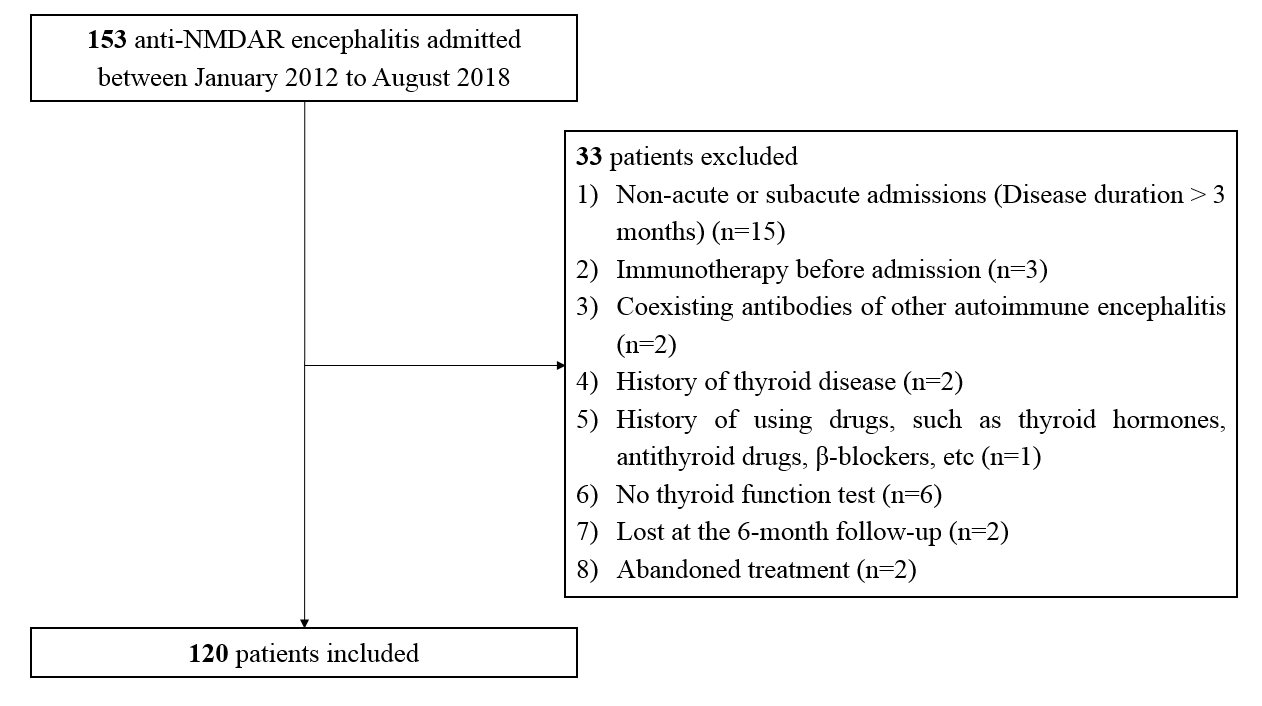

Supplement: Supplementary Figure 1 — Patient enrollment flow chart. [file Image_1.tif]
